# Editorial Comment to Robot‐assisted laparoscopic partial nephrectomy for horseshoe kidney: A case report

**DOI:** 10.1002/iju5.12121

**Published:** 2019-09-12

**Authors:** Takamitsu Inoue

**Affiliations:** ^1^ Department of Urology Akita University School of Medicine Akita Japan

Molina and Gill reported the first case of laparoscopic partial nephrectomy (LPN) for horseshoe kidney (HSK) by the retroperitoneal approach in 2003.^1^ The first case of robotic‐assisted partial nephrectomy (RAPN) for HSK was reported from the UK in 2017^2^ and the second case was reported from Japan.^3^ In the present case report, Fujihara *et al*. reported the third successful case of RAPN for HSK.^4^ The renal mass, which had a diameter of 2 cm in the lateral mid‐pole of the left moiety, was resected using the transperitoneal RAPN procedure.

As discussed by the authors in the present case report, there are three anatomically challenging issues in LPN or RAPN for HSK: (i) high variability of the vasculature; (ii) the presence of the isthmus that could cause reduced mobility of the moiety; and (iii) renal pelvic and ureteral passage in front of the isthmus. Therefore, in the contemporary environment of imaging technology and robotic surgery, it is essential to reconstruct a detailed preoperative three‐dimensional (3D) image for each patient to determine whether the trans‐ or retroperitoneal approach should be used and to determine the necessity of isthmusectomy based on the site of the tumor before and during the surgical treatment of HSK. The 3D reconstruction images have been published in 6 of 10 previous case reports of LPN or RAPN for HSK. However, there have been only three recent case reports (including the present case) in which the contour and positional relationships among the renal parenchyma, tumor, and renal arteries were clearly identified.^2^, ^4^, ^5^

In addition to the previous detailed discussion in the LPN era, there may be slight additional caution in the RAPN era. Tsivian *et al*.^6^ suggested that the choice of trans‐ or retroperitoneal approach depends on tumor location such as anterior, anterolateral, and isthmic locations that are suitable for the transperitoneal approach, whereas posterior and posterolateral tumors are suitable for the retroperitoneal approach; the same recommendations are applicable in the RAPN era. There has been no previous report on isthmusectomy in LPN or RAPN for HSK. Around the vicinity of the isthmus, the transperitoneal approach is suitable for a large operative area^5^ and the retroperitoneal approach should be cautious in cases where the robot arm may not extend to the isthmus.

## Conflict of interest

The author declares no conflict of interest.
